# Analysis of Metabolic Markers in Patients with Chronic Heart Failure before and after LVAD Implantation

**DOI:** 10.3390/metabo11090615

**Published:** 2021-09-09

**Authors:** Marion S. Hilse, Tom Kretzschmar, Rudin Pistulli, Marcus Franz, Tarek Bekfani, Daniela Haase, Sophie Neugebauer, Michael Kiehntopf, Jan F. Gummert, Hendrik Milting, P. Christian Schulze

**Affiliations:** 1 Department of Internal Medicine I, Division of Cardiology, University Hospital Jena, 07747 Jena, Germany; Marion.hilse@klinikumfrankfurt.de (M.S.H.); tom.kretzschmar2@med.uni-jena.de (T.K.); Marcus.Franz@med.uni-jena.de (M.F.); Daniela.Haase@med.uni-jena.de (D.H.); 2 Department of Cardiology I—Coronary and Peripheral Vascular Disease, Heart Failure, Münster University Hospital, 48149 Münster, Germany; Rudin.Pistulli@ukmuenster.de; 3 Department of Internal Medicine I, Division of Cardiology, Angiology and Intensive Medical Care, University Hospital Magdeburg, 39120 Magdeburg, Germany; tarek.bekfani@med.ovgu.de; 4 Department of Clinical Chemistry and Laboratory Diagnostics, University Hospital Jena, 07747 Jena, Germany; Sophie.Neugebauer@med.uni-jena.de (S.N.); Michael.Kiehntopf@med.uni-jena.de (M.K.); 5 Heart and Diabetes Center NRW, 32545 Bad Oeynhausen, Germany; jgummert@hdz-nrw.de (J.F.G.); hmilting@hdz-nrw.de (H.M.)

**Keywords:** DCM, ICM, LVAD, mass spectrometry, metabolomics

## Abstract

Chronic heart failure (HF) is a clinical syndrome characterized by functional impairments of the myocardium. Metabolic and clinical changes develop with disease progression. In an advanced state, left ventricular assist devices (LVADs) are implanted for mechanical unloading. Our study aimed to assess the effects of LVAD implantation on the metabolic phenotypes and their potential to reverse the latter in patients with advanced HF. Plasma metabolites were analyzed by LC–MS/MS in 20 patients with ischemic cardiomyopathy (ICM), 20 patients with dilative cardiomyopathy (DCM), and 20 healthy controls. Samples were collected in HF patients before, 30 days after, and >100 days after LVAD implantation. Out of 188 measured metabolites, 63 were altered in HF. Only three metabolites returned to pre-LVAD concentrations 100 days after LVAD implantation. Pre-LVAD differences between DCM and ICM were mainly observed for amino acids and biogenic amines. This study shows a reversal of metabolite abnormalities in HF as a result of LVAD implantation. The etiology of the underlying disease plays an essential role in defining which specific metabolic parameter is altered in HF and reversed by LVAD implantation. Our findings provide a detailed insight into the disease pattern of ICM and DCM and the potential for reversibility of metabolic abnormalities in HF.

## 1. Introduction

Cardiovascular mortality is the most common cause of death in the western hemisphere, with 3.9 million deaths per year in Europe [1]. Heart failure (HF) describes the inability of the heart to meet the metabolic needs of the body, with congestion and exercise intolerance as the main symptoms [2]. HF leads to irreversible cardiac muscle damage and myocardial remodeling, associated with local and systemic metabolic abnormalities and end-organ damage [3,4,5,6,7]. The risk of HF for subjects over 55 years is 33% for men and 29% for women [8], with a five-year survival rate of 51.5% [9].

The implantation of a left ventricular assist device (LVAD) has become an established non-pharmacologic therapy for patients with advanced HF waiting for heart transplantation or as destination therapy [10,11,12]. Advanced HF is in part characterized by reduced LVEF ≤ 30% and constant symptoms of heart failure (NYHA class III–IV) [13], while end-stage HF is in part defined by multiple and irreversible organ damage [14]. Patients with LVAD support have an increased first-year survival rate and an overall improved quality of life [15,16].

Since cardiac and systemic metabolic abnormalities are a hallmark of advanced HF, we hypothesized that mechanical unloading through LVAD implantation leads to changes and partial reversibility in metabolic parameters. These include acylcarnitines (ACs), glycerophospholipids (GPs), sphingomyelins (SMs), amino acids (AAs), and biogenic amines (BAs) that are altered in patients with advanced HF compared to controls.

## 2. Results

### 2.1. Baseline Characteristics

The control group included 13 women and 7 men with an average age of 66.3 ± 6.7 years (median 66.0 years). None of the control patients had a diagnosed cancerous disease, chronic inflammatory disease, chronic obstructive pulmonary disease (COPD) or were immunosuppressed. The heart failure (HF) subgroup included 20 patients with dilative cardiomyopathy (DCM) and 20 patients with ischemic cardiomyopathy (ICM).

DCM is characterized by an enlarged LV with reduced systolic function. ICM is caused by cardiovascular circulatory disorder leading to irreversible cardiac muscle damage and remodeling. The DCM group consisted of 4 women and 16 men with an average age of 58.2 ± 12.4 years (median 58.0 years). The ICM group included 6 women and 14 men, with an average age of 60.2 ± 10.1 years (median 60.5 years). Blood samples were collected before LVAD implantation and as follow-up 30 days and over 100 days after LVAD implantation. Demographic data and cardiovascular risk factors were collected. In addition, functional and morphological parameters such as LV-EF and LVEDD before and after LVAD implantation were measured (Table 1).

**Figure 1:**
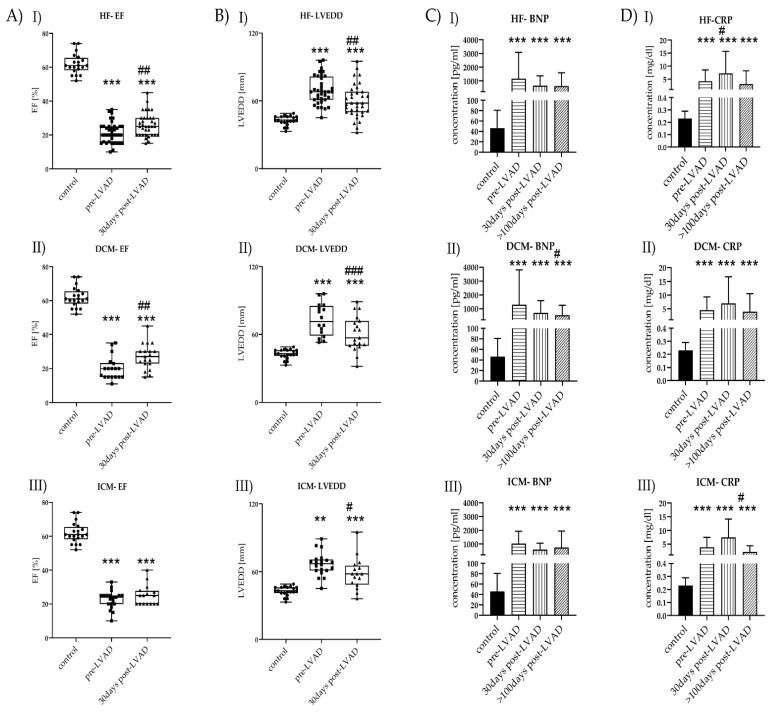
Time course of laboratory and clinical markers before and after LVAD implantation of HF (I), DCM (II), and ICM (III)*. (***A**) LV-EF levels of control (*n* = 20) vs. HF (*n* = 40), control vs. DCM (*n* = 20), and control vs. ICM (*n* = 20), all with pre-LVAD and 30 days post-LVAD. Depicted are min, max, median, and single values. Significance was calculated with a *t*-test or the Mann–Whitney U test. * indicates significant to control (*** = *p* < 0.001). # indicates significant to pre-LVAD (## = *p* < 0.01). (**B**) LVEDD levels of control vs. HF, control vs. DCM, and control vs. ICM, all with pre-LVAD and 30 days post-LVAD. Depicted are min, max, median, and single values. Significance was calculated with a *t*-test or the Mann–Whitney U test * indicates significant to control (** = *p*<0.01, *** = *p* < 0.001). # indicates significant to pre-LVAD (# = *p* < 0.05, ## = *p* < 0.01, ### = *p* < 0.001). (**C**) BNP levels of control vs. HF, control vs. DCM, and control vs. ICM all with pre-LVAD, 30 days post-LVAD, and >100 days post-LVAD. Depicted are mean values and standard deviation. Significance was calculated with a *t*-test or the Mann–Whitney U test * indicates significant to control (*** = *p* < 0.001). # indicates significant to pre-LVAD (# = *p* < 0.05). (**D**) CRP levels of control vs. HF, control vs. DCM, and control vs. ICM, all with pre-LVAD, 30 days post-LVAD, and >100 days post-LVAD. Depicted are mean values and standard deviation. Significance was calculated with a *t*-test or the Mann–Whitney U test. * indicates significant to control (*** = *p* < 0.001). # indicates significant to pre-LVAD LVAD (# = *p* < 0.05).

HF patients showed reduced LV-EF compared to healthy controls, which slightly improved after LVAD implantation (Figure 1(AI)). DCM patients showed a better response to the therapy regarding LV-EF, in contrast to ICM patients (Figure 1(AII–III)). LVEDD was increased in pre-LVAD HF compared to controls and showed minor improvement after LVAD implantation (Figure 1(BI)). Reduction of post-LVAD LVEDD was equally observed in DCM and ICM patients (Figure 1(BII–III)). LV-EF and LVEDD data >100 days post-LVAD were not available.

### 2.2. Laboratory Parameters

Standard laboratory parameters were measured to monitor patient status before and after LVAD implantation (Table 2) and controls.

HF patients showed increased leukocyte counts before LVAD, which decreased >100 days post-LVAD. BNP showed the highest levels before LVAD implantation but was decreased post-LVAD (Figure 1(CI–III)). CRP concentrations were highest in HF patients before LVAD treatment (Figure 1(DI)). CRP values remained high during the surveyed period, with their highest peak 30 days post-LVAD. CRP was increased pre-LVAD in ICM patients and remained increased over the observed period. CRP was significantly lower >100 days post-LVAD ICM. Further, CRP reached levels comparable to pre-LVAD levels after >100 days post-LVAD but was still increased compared to controls (Figure 1(DI–III)).

### 2.3. Metabolomic Profiles of Subjects with HF Pre-LVAD Compared to Controls

**Figure 2:**
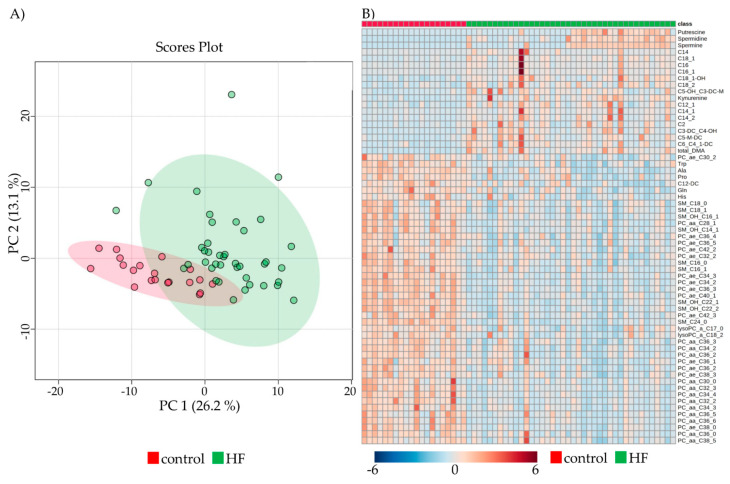
Comparison of metabolic parameters between control and HF. (**A**) Principal component analysis of all patients and control (red) vs. pre-LVAD HF (green), depicting overlapping and dispersing patients with four outliers. (**B**) Heatmap visualization of the 63 significantly altered metabolites between control and HF. Colors indicate the concentration of the respective metabolite. Columns signify samples, and rows metabolites.

PCA showed distinct and overlapping metabolites of control and HF, with a visible differentiation between the two groups (Figure 2A).

Out of the 188 determined metabolites, 33.5% (*n* = 63) were altered in HF pre-LVAD compared to controls after Bonferroni correction (Figure 2B). Classification of the metabolites into groups revealed 15/40 ACs (37.5%, 1 decreased, 14 increased), 5/20 AAs (25%, all decreased), 5/13 BAs (38.5%, all increased), 29/90 GPs (32.2%, all decreased), and 9/15 SMs (60%, all decreased) showed variations pre-LVAD implantation.

When compared to the control, DCM and ICM showed a different level of alteration for some metabolites. For example, AC C14 was increased in HF but only passed the Bonferroni threshold in ICM and not in DCM. Therefore, the increase of C14 was more prominent in ICM; even so, no significant difference was detected when ICM and DCM were compared directly. Table 3 shows that LVAD implantation affected ACs and SMs in ICM patients, while AAs and PCs were more affected in DCM.

Only three out of 63 changed metabolites returned to control level after more than 100 days post-LVAD: AC C2, AS proline (Pro), and PC lysoPC a C17:0. C2 was significantly decreased 30 days post-LVAD HF (*p* = 0.05) compared to pre-LVAD HF. Additionally, Pro (*p* < 0.001) and lysoPC a C17:0 (*p* = 0.001) were significantly increased >100 days post-LVAD HF compared to pre-LVAD HF.

### 2.4. Comparison between DCM and ICM at Baseline and during Follow-Up

**Figure 3:**
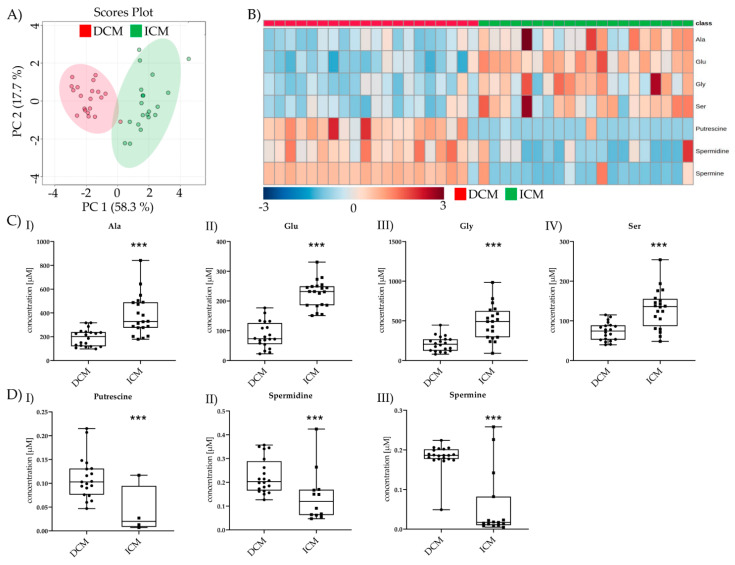
Comparison of significant metabolic parameters of DCM and ICM pre-LVAD. (**A**) PCA of pre-LVAD DCM vs. pre-LVAD ICM significantly different metabolites. (**B**) Heatmap visualization of the seven metabolites significantly different between DCM and ICM pre-LVAD. (**C**) I–IV Amino acid levels of pre-LVAD DCM vs. pre-LVAD ICM. Depicted are min, max, median, and single values. Significance was calculated with a *t*-test) or the Mann–Whitney U test). * indicates significant to DCM (*** = *p* < 0.001) (**D**) I–III Biogenic amine levels of pre-LVAD DCM vs. pre-LVAD ICM. Depicted are min, max, median, and single values. Significance was calculated with a *t*-test or the Mann–Whitney U test. * indicates significant to DCM (*** = *p* < 0.001).

Seven metabolites were changed between pre-LVAD DCM and pre-LVAD ICM. PCA and Heatmap allowed visualizing the differences between the two groups (Figure 3A,B). Four AAs (Ala, Glu, Gly, Ser) were significantly lower in pre-LVAD DCM compared to pre-LVAD ICM (Figure 3(CI–IV)); three BAs (putrescine, spermidine, spermine) were increased in pre-LVAD DCM (Figure 3(DI–III)).

**Figure 4:**
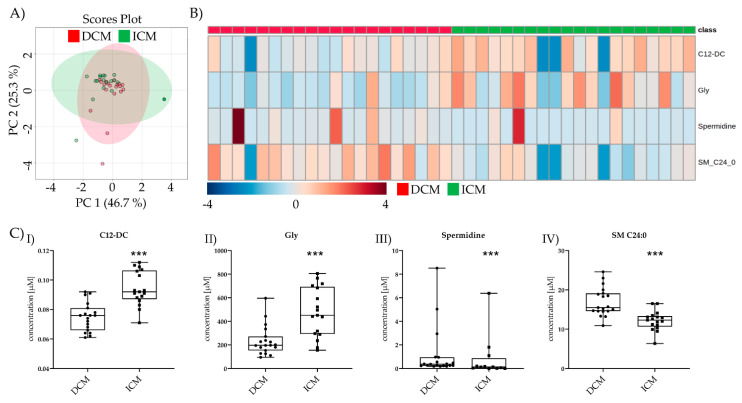
Comparison of significant metabolic parameters of DCM and ICM >100 days post-LVAD. (**A**) PCA of the significantly different metabolites >100 days post-LVAD DCM vs. >100 days post-LVAD ICM. (**B**) Heatmap visualization of the four metabolites significantly different between DCM and ICM >100 days post-LVAD. (**C**) I–IV C12-DC, Gly, spermidine, and SM C24:0 levels >100 days post-DCM vs. >100 days post-LVAD ICM. Depicted are min, max, median, and single values. Significance was calculated with a *t*-test) or the Mann–Whitney U test). * indicates significant to DCM (*** = *p* < 0.001).

After >100 days post-LVAD, only four metabolites were altered between >100 days post-LVAD DCM and >100 days post-LVAD ICM (Figure 4A,B). Gly and spermidine were still increased after >100 days. Putrescine was no longer detectable in ICM and was excluded. The previously unchanged metabolite C12-DC was decreased in >100 days post-LVAD DCM, and SM 24:0 was increased >100 days post-LVAD DCM both compared to >100 days post-LVAD ICM (Figure 4(CI–IV)).

**Figure 5:**
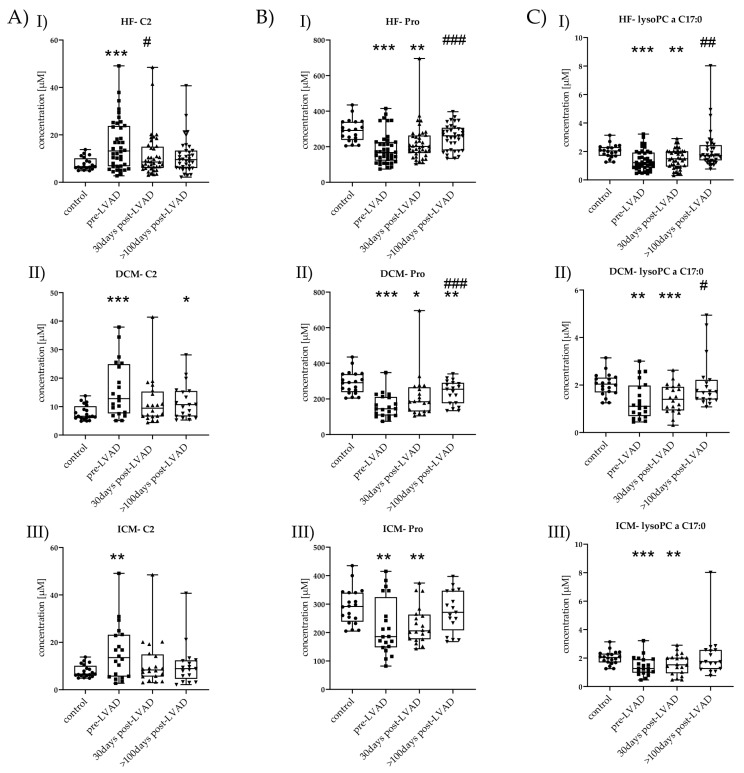
Time-course changes of C2, Pro, and lysoPC a C17:0 in HF, DCM, and ICM. (**A**) I–III C2 levels of control vs. HF, DCM, and ICM with pre-LVAD, 30 days post-LVAD, and >100 days post-LVAD. Depicted are min, max, median, and single values. Significance was calculated with a *t*-test or the Mann–Whitney U test * indicates significant to control (* = *p* < 0.05, ** = *p* < 0.01, *** = *p* < 0.001). # indicates significant to pre-LVAD (# = *p* < 0.05). (**B**) I–III Pro levels of control vs. HF, DCM, and ICM all with pre-LVAD, 30 days post-LVAD, and >100 days post-LVAD. Depicted are min, max, median, and single values. Significance was calculated with a *t*-test or the Mann–Whitney U test * indicates significant to control (* = *p* < 0.05, ** = *p* < 0.01, *** = *p* < 0.001). # indicates significant to pre-LVAD (### = *p* < 0.001). (**C**) I–III lysoPC a C17:0 levels of control vs. HF, DCM, and ICM all with pre-LVAD, 30 days post-LVAD, and >100 days post-LVAD. Depicted are min, max, median, and single values. Significance was calculated with a *t*-test or the Mann–Whitney U test * indicates significant to control (** = *p* < 0.01, *** = *p* < 0.001). # indicates significant to pre-LVAD (# = *p* < 0.05, ## = *p* < 0.01).

Only three metabolites (C2, Pro, lysoPC a C17:0) returned to normal concentrations >100 days post-LVAD and thus might be useful as potential biomarkers for monitoring LVAD. Therefore, we considered these metabolites separately to identify detailed expressional differences between DCM and ICM during follow-up (Figure 5).

In DCM, C2 was increased pre-LVAD (15.98 ± 9.65, *p* = 0.001); it was not increased 30 days post-LVAD (11.58 ± 8.21, *p* = 0.07) but increased again >100 days post-LVAD (11.79 ± 6.08, *p* = 0.02) compared to control (7.83 ± 2.61) (Figure 5(AII)). C2 showed a tendency to reduction with time progression.

Pro was lower pre-LVAD DCM (160.45 ± 64.74, *p* < 0.001), 30 days post-LVAD DCM (215.4 ± 127.0, *p* = 0.02), and >100 days post-LVAD DCM (237.47 ± 65.02, *p* = 0.01) but also showed an increase over time compared to control (291.05 ± 61.73). Pro was significantly increased >100 days post-LVAD (*p* < 0.001) compared to pre-LVAD DCM (Figure 5(BII)).

LysoPC a C17:0 was decreased pre-LVAD DCM (1.35 ± 0.76, *p* = 0.003) and 30 days post-LVAD DCM (1.42 ± 0.60, *p* = 0.001). The reduction was no longer observed >100 days post-LVAD (2.05 ± 1.05, *p* = 0.2) compared to control (2.02 ± 0.46). Additionally, its concentration >100 days post-LVAD (*p* = 0.01) was significantly higher compared to that pre-LVAD DCM (Figure 5(CII)).

In ICM, C2 was also increased pre-LVAD (15.77 ± 11.47, *p* = 0.008), but no longer 30 days post-LVAD (11.40 ± 10.06, *p* = 0.14) and >100 days post-LVAD (10.55 ± 8.84, *p* = 0.48) compared to control (Figure 5(AIII)).

Pro was decreased pre-LVAD ICM (219.2 ± 97.25, *p* = 0.01) and 30 days post-LVAD (228.5 ± 65.10, *p* = 0.004) but no longer >100 days post-LVAD (274.59 ± 70.73, *p* = 0.47). Pro level constantly increased after LVAD implantation till it reached a non-significant level (Figure 5(BIII)). LysoPC a C17:0 was decreased pre-LVAD ICM (1.42 ± 0.65, *p* = 0.001) and 30 days post-LVAD (1.57 ± 0.71, *p* = 0.03) but was no longer altered >100 days post-LVAD (2.19 ± 1.59, *p* = 0.55) (Figure 5(CIII)).

### 2.5. Correlations of Metabolites with Clinical and Standard Laboratory Parameters

We analyzed the correlation between the three reversible metabolites (C2, Pro, lysoPC a C17:0) pre-LVAD, 30 days post-LVAD, and >100 days post-LVAD with CRP and BNP at the same respective time point. We detected a positive correlation of 30 days post-LVAD C2 with 30 days post-LVAD CRP and BNP (ρ = 0.286, *p* = 0.03 for CRP, ρ = 0.348, *p* = 0.015 for BNP) but none for any other tested condition. No correlations between Pro and either CRP or BNP were determined. LysoPC a C17:0 showed a negative correlation for pre-LVAD and 30 days post-LVAD and CRP (ρ = −0.350, *p* = 0.029 for pre-LVAD, ρ = −0.565, *p* < 0.001 for 30 days post-LVAD). No correlations were detected for LysoPC a C17:0 and BNP.

We further determined the correlations of the initially altered 63 metabolites as well as of CRP and BNP. An overview of the correlations is depicted in supplementary Tables S1 and S2. Detailed correlations and significances are listed in supplementary Table S3.

Additional correlations were calculated for genders, but no significance was detected. Furthermore, the correlation regarding age was performed. CRP showed a positive correlation for 30 days post-LVAD and age (ρ = 0.384, *p* = 0.014). A positive correlation was also assessed for 30 days post-LVAD BNP and age (ρ = 0.322, *p* = 0.046).

## 3. Discussion

Our data showed that LVAD implantation improves functional and laboratory parameters as well as inflammatory markers in HF patients [17,18].

Further, patients with chronic HF displayed severely altered fatty acid metabolism characterized by increased plasma concentrations of ACs, BAs, and decreased concentrations of GPs, SMs, and AAs compared to healthy controls. After LVAD implantation, ACs returned to control levels in ICM but not in DCM patients. GPs and SMs concentrations improved after LVAD implantation in DCM but not in ICM patients. Out of the measured AAs, only Pro level recovered >100 days post-LVAD in ICM patients. Of BAs, only spermine showed a reduction to the control level in ICM patients >100 days post-LVAD but remained overall increased in the HF group. In part, these data concur with the results of Haase et al., who identified decreased phosphatidylcholines (PCs) in patients with symptomatic aortic stenosis prior to valve replacement [19].

Long-chain fatty acids are transported through the mitochondrial membrane by carnitine acylcarnitine transferase as ACs. Through β-oxidation [20], they are substrates for mitochondrial ATP generation. Fatty acid β-oxidation is impaired in HF patients, leading to accumulation of ACs in tissues and in the circulation [21,22]. Ruiz et al. specified that both long-chain ACs and those with hydroxy (OH–AC) and dicarboxylic groups (DC–AC) were increased in HF patients [16], which we could confirm in the current study. The increased number of circulating long-chain ACs is associated with reduced functional status and increased mortality, which are affected by LVAD implantation [23]. Ahmad et al. particularly emphasized that ACs C16:0, C18:1, and C18:2, were significantly elevated in advanced HF patients. More than 90 days after the implantation of a mechanical cardiac support system, a decrease in ACs was observed [23]. Further, in our analysis after LVAD implantation, previously increased C16:0 and C18:1 ACs decreased within the first 30 days. C18:2 even returned to normal levels 30 days post-LVAD HF, which, however, was no longer observed >100 days post-LVAD. Thus, our data imply no significant long-term reversibility and benefits from LVAD implantation regarding ACs.

No analysis has addressed the reversibility of AC concentrations separately in patients with ICM and DCM after implantation of a mechanical cardiac support system. Previous studies showed that the increase of circulating ACs in advanced HF patients is reversible after LVAD implantation [23]. The more precise differentiation between ICM and DCM in the present study showed a reversible course of ACs in certain cases. The reversibility was more pronounced in ICM compared to DCM patients. The individual analysis of metabolite concentrations showed that C14, C18:2, and C6:C4:1-DC were significantly increased in pre-LVAD ICM but not in pre-LVAD DCM compared to healthy controls. C12-DC, C14:1, C16, and C16:1 were significantly altered in pre-LVAD DCM but not in pre-LVAD ICM. C14, C18:2, C6:C4:1-DC C18:1, C18:1-OH, C18:2, C5-M-DC returned to normal levels >100 days post-LVAD ICM. Most of the ACs in DCM remained altered after LVAD implantation. Further research is necessary to determine why these ACs are predominantly affected in ICM compared to DCM. When compared directly, no AC showed a significant change between DCM and ICM. Still, the fact that some ACs are more prominently altered in ICM or DCM when compared to the control could be interesting for the specific metabolic characterization of ICM and DCM.

Only AC C2 returned to control concentration >100 days post-LVAD HF and showed similar decreasing behavior in DCM and ICM. There seems to be no exclusive connection between this specific AC and HF patients in general. C2 is mainly listed as one out of many ACs altered by HF and affected by specific treatment methods [17,19,21,24]. Further investigations are necessary to elucidate the causality between the observed altered C2 concentration pre- and post-LVAD implantation.

PCs, together with phosphatidylethanolamine and phosphatidylserine, is the main components of biological membranes. PCs are also found in mitochondrial membranes and are mainly synthesized in the endoplasmic reticulum [25]. Previous studies reported a significant reduction of PCs in HF patients compared to healthy controls [17,26]. In non-ischemic cardiomyopathy, Mueller-Hennessen et al. described that decreased PC concentrations of C16:0 and C18:2 were accompanied by increased HF severity (NYHA I vs. II vs. III) [26]. Interestingly, the reduction in PC concentration pre-LVAD was more pronounced in DCM than in ICM compared to control. After LVAD implantation, PC levels increased again, with significant changes mainly observed in the DCM group.

Of all the measured PCs and lysoPCs, only lysoPC a C17:0 returned to normal levels after LVAD implantation in HF. LysoPCs are derivates of phosphatidylcholines and catalyzed by phospholipase A_2_. They are mostly known for their involvement in atherosclerosis [27,28,29,30,31]. The potential of lysoPCs as biomarkers is divisive [32]. Some studies reported a negative correlation between circulating plasma lysoPCs and cardiovascular disease [33,34,35], while others identified some PCs as potential biomarkers for myocardial infarction [36]. The findings of our study concur with those of Ward-Caviness et al. [36] and Haase et al. [19], suggesting lysoPCs as a potential prognostic markers for HF patients.

SMs belong to the group of phospholipids as well as to that of sphingolipids. Sphingolipids are synthesized in the endoplasmic reticulum and are located in cell membranes. They are considered important mediators of inflammation; in addition, they play an essential role in cell cycle regulation, stress responses, proinflammatory signaling pathways, and cell migration [37]. SMs are significantly decreased in chronic HF patients compared to healthy controls [26,38]. Polzin et al. investigated cardiomyopathies of ischemic origin and found that reduced SM concentrations correlated with reduced LV-EF and higher NYHA stage [39]. Significant long-term increases in plasma SM after LVAD implantation were observed for SM (OH) C14:1, SM (OH) C22:1, SM C16:0, and SM C24:0 in our study. When the ICM and DCM groups were considered separately, significant reversibility of SM C20:2 was only found in the DCM group. No significant changes were identified in the ICM group. However, tendencies towards increased concentrations after LVAD implantation were observed in both groups.

Amino acid metabolism is altered in subjects with HF [40,41,42]. AAs were the primary metabolites that were significantly different between DCM and ICM pre-LVAD. The concentrations of Glu, Gly, and Ser were not changed in pre-LVAD ICM compared to control, while those of Glu and Ser were significantly decreased in pre-LVAD DCM. Gly showed a tendency to a decreased level. That indicates that alterations in amino acid metabolism are mainly observed in DCM and are more severe. Amino acids seem to be less affected in ICM patients. Our data indicate that the levels of certain amino acids are important criteria for the metabolic differentiation of DCM and ICM and could be potential biomarkers.

Only one amino acid returned to control concentrations >100 days post-LVAD HF. Proline is an important player in glutamate metabolism and, as such, is involved in different signaling pathways, including metabolic reprogramming [43,44], senescence [45], and apoptosis [46,47]. Wang et al. recently showed that Pro acts a cardioprotective agent by reducing reactive oxygen species in vitro [48]. This finding supports and potentially explains the results of Fan et al., who detected an increased activation of the Pro metabolic pathway in patients with coronary artery disease [49]. In our study, DCM and ICM patients showed reduced levels of Pro before LVAD implantation. This reduction potentially indicates negatively altered glutamate metabolism; that, in turn, hints that Pro’s cardioprotective effects were reduced and potentially insufficient to counteract associated detrimental effects. This further underlines the severity of untreated ICM and DCM. After LVAD implantation, the metabolic changes were in part reversed, resulting in normalized Pro concentrations and overall improvement of quality of life. More studies are required to understand and verify the definitive role of Pro as a cardioprotective amino acid in different HF states. However, the possibility of Pro as a potential prognostic marker is valuable and supported by our data.

Spermidine and spermine are important proteins for cell growth, and their expression is known to decline with progressing age [50]. The observed increased expression in DCM but not ICM could again be a counter mechanism of the failing myocardium in the more severe DCM. At this point, we cannot define the definitive role of spermidine and spermine as potential differentiation markers between DCM and ICM. Further studies are necessary to elucidate the respective roles and to exclude any age-related effect.

Our study has some limitations. Even though three different time points were available and used for sample collection, our patient cohort of just 20 patients per group (DCM and ICM) was still small compared to other studies [21,51]. Furthermore, heart failure with reduced ejection fraction (HFrEF) is more prominent in men, explaining the observed gender differences in the respective groups. Larger control and patient cohorts as well as a comparable gender distribution could improve the quality of our data. In addition, an even more advanced time point after LVAD implantation could shed more light on the metabolic behavior of previously unchanged metabolic parameters. This could further elucidate the potential of specific metabolites as prognostic biomarkers or drug targets.

## 4. Material and Methods

### 4.1. Patient Cohort

A total of 40 patients (20 DCM, 20 ICM) with advanced HF who underwent LVAD implantation between June 2008 and May 2015 were recruited into the study. The patients in the control group (control, *n* = 20) were recruited from the outpatient center at the University Hospital Jena. Blood samples were taken from all individuals, and serum and plasma were stored at −80 °C until analysis. None of the samples were used in previous studies. Inclusion criteria were advanced HF (New York Heart Association (NYHA) (III–IV) with left ventricular ejection fraction (LV-EF) below 35% and the implantation of an LVAD. Patients had to be older than 18 years, and a written consent form had to be present. Patients with a cardiovascular event two weeks before the study or those who suffered from an acute infection or did not have a written consent form were excluded from the study. Blood samples were collected at three different time points: immediately before implantation (pre-LVAD), approximately 30 days (mean 30.9 ± 14.5 days, median 26 days) after LVAD implantation (30 days post-LVAD), and more than 100 days after LVAD implantation (>100 days post-LVAD). The Ethics Committee of the University Hospital Jena approved the study (processing number 4768 04/16). Written consent forms were obtained from all patients included in the study (*n* = 60).

### 4.2. Laboratory and Mass Spectrometric Analysis

Laboratory parameters such as number of platelets, erythrocytes, leucocytes, levels of total cholesterol, triglycerides, asparagine aminotransferase (ASAT), alanine aminotransferase (ALAT), creatinine, urea, C-reactive protein (CRP), glucose, and b-natriuretic peptide (BNP), LV-EF and left ventricular end-diastolic diameter (LVEDD) were measured routinely at the Heart and Diabetes Center NRW in Bad Oeynhausen (patients) and the University Hospital Jena (control). Six analytical classes of metabolites (90 GPs, 40 ACs, 21 AAs, 21 BAs, 15 SMs, and 1 sugar) were measured in EDTA plasma samples and quantified with the AbsoluteIDQ^®^ p180 kit (Biocrates Life Science AG, Innsbruck, Austria) according to the manufacturer’s protocol. An API4000 liquid chromatography–tandem mass spectrometry (LC–MS/MS) system (AB Sciex, Framingham, MA, USA) was used for measurement. The device was additionally equipped with an electrospray ionization source and a CTC PAL autosampler (CTC Analytics AG, Zwingen, Switzerland) and the Analyst 1.6.2. Software (AB Sciex). Calibration curves, quality controls, and samples were evaluated with the MetIQ software package, which is an integrated part of the used kit. Three replicates of a reference sample served for data normalization on the same plate, and the concentrations were exported for the following statistical analysis.

### 4.3. Data Analysis and Statistics

The Metaboanalyst software (version 4.0) was used for statistical analysis and the graphical representation of large datasets. Statistical analysis was performed with SPSS. Data were depicted either as mean value ± standard deviation or as median with single values, min, and max values. Normal distribution was calculated with the Kolmogorov–Smirnov test. First, an analysis of variance (ANOVA) for more than two samples was performed to compare the three groups (control, DCM, and ICM). Single-factor analysis of variance (parametric) was applied for normal distribution, otherwise the Kruskal–Wallis test (non-parametric) was used. Also, the *t*-test and the Mann–Whitney U test were performed for significance testing (*p* < 0.05). A Bonferroni correction was done to determine the significance threshold for group differences and the adjustment for multiple testing. The correlation coefficient was calculated with Spearman´s rank correlation (ρ). Significance of Spearman’s ρ was calculated two-tailed for the preLVAD condition and one-tailed for 30 days post-LVAD and >100 days post-LVAD because of the effect assumption of LVAD implantation.

## 5. Conclusions

Our study provides a comprehensive analysis of metabolic changes pre- and post-LVAD implantation in DCM and ICM patients. It enabled a direct comparison over a well-documented period. The beneficial effects of LVAD implantation were demonstrated by the improvement of different laboratory parameters and, further, by changes in individual metabolites. Our data thus provide new insights into HF progression and reversibility. The generation of a detailed metabolic profile shows its value for progress monitoring and potential identification of new biomarkers and drug targets. In the future, disease-dependent and -specific metabolic profiles could improve disease monitoring and survey progression after LVAD implantation and other forms of cardiomyopathy treatment.

## Figures and Tables

**Table 1:** Clinical and demographic data of the patients.

	Controls (*n* = 20)	DCM (*n* = 20)	ICM (*n* = 20)	*p* Value
Demographics				
Female/male (w%)	13/7 (65%)	4/16 (20%)	6/14 (30%)	0.010
Age (years)	65.5 (9)	58.0 (19)	60.5 (16)	0.062
BMI (kg/m^2^)	25.2 (4.8)	27.4 (5.3)	24.6 (6.2)	0.091
LV characteristics				
LV-EF (%) (after LVAD)	61.0 (7)	20.0 (7) * 27.0 (7) **	24.0 (5) * 25.0 (8) **	<0.0001 0.443
LVEDD (mm) (after LVAD)	43.0 (26.3)	71.5 (26.3) * 57.0 (22) **	67.0 (9.8) 58.0 (16.8) **	<0.0001 0.667
CV risk factors				
Type 2 diabetes	2	5	1	0.246
Arterial Hypertension (≥140/90 mmHg)	10	8	11	0.726
Overweight (BMI > 25)	11	16	10	0.127

Data are depicted as median and Interquartile Range (median (IQR)). * significant to pre-LVAD, ** significant to post-LVAD. Significance was calculated with Kruskal–Wallis test. (*p* < 0.05). BMI = body mass index, LV-EF = left ventricular ejection fraction, LVEDD = Left Ventricular End-Diastolic Diameter, CV = cardiovascular.

**Table 2:** Laboratory parameters.

	Controls	Heart Failure			
		Pre-LVAD	30 Days Post-LVAD	>100 Days Post-LVAD	*p* Value
Leucocytes (10^9^/L)	6.1 (1.9)	8.5 (5.4) °^,%^	9.6 (4.6) ^§^	7.4 (4.0)	<0.0001 *
Cholesterol (mg/dL)	232.4 (60.7)	162.0 (82) °	169.0 (72)	198.5 (72)	<0.001 *
Triglycerides (mg/dL)	118.6 (71.3)	97.5 (78.8) ^#,%^	113.5 (82.8)	138.5 (140)	0.417
ASAT (U/L)	28.0 (5.8)	31 (24)	30 (20.3)	28 (13)	0.461
ALAT (U/L)	22.5 (10)	24 (22) ^#^	19.5 (21.8)	21.5 (14.3)	0.475
CRP (mg/dL)	0.2 (0.03)	3.1 (2.9) °^,%^	4.3 (7.5) ^§^	0.84 (2.1)	<0.0001 *
Creatinine (mg/dL)	0.78 (0.2)	1.3 (0.8) °	1.1 (0.6) ^§^	1.5 (1)	<0.001 *
Urea (mg/dL)	34.0 (13.3)	52.0 (43) °	40.5 (46)	45.0 (41.5)	0.006 *
BNP (pg/mL)	35.5 (31.3)	612.0 (1104.9) °^,%^	392.0 (438.2) °	354.7 (445.9) °	<0.0001 *

Data are depicted as median and Interquartile Range (median (IQR)). Significance was calculated with ANOVA/Kruskal–Wallis test and indicated with *. Significances calculated with *t*-test and Mann–Whitney U test are indicated with: ° = *p* < 0.05 pre-LVAD compared to control, # = *p* < 0.05 pre-LVAD compared to 30 days post-LVAD, ^%^ = *p* < 0.05 pre-LVAD compared to >100 days post-LVAD, ^§^ = *p* < 0.05 30 days post-LVAD compared to >100 days post-LVAD, ASAT = Asparagine Aminotransferase, ALAT = Alanine Aminotransferase, BNP = Brain Natriuretic Peptide, CRP = C- reactive protein.

**Table 3:** Summarized list of altered metabolites of control vs. HF.

Metabolite Class	Pre-LVAD		>100 Days Post-LVAD	
	DCM	ICM	DCM	ICM
AC	8	7	7	1
AA	9	1	2	0
BA	4	2	7	1
PC	29	18	19	15
SM	8	10	6	5

Individual metabolites of DCM and ICM were compared to control and tested for significant changes. Depicted are the numbers of metabolites for each metabolite class that passed the Bonferroni threshold.

## Data Availability

All data, tables and figures in this manuscript are original. The data presented in this study are available in the article or Supplementary Material.

## Supplementary Materials

The following are available online https://www.mdpi.com/article/10.3390/metabo11090615/s1, Table S1: Correlations overview between metabolites and CRP at the three examined time points, Table S2: Correlations overview between metabolites and B at the three examined time points, Table S3: Detailed correlations and significance.
