# Supplementary material for: Analysis of Metabolic Markers in Patients with Chronic Heart Failure before and after LVAD Implantation
Source: Metabolites. 2021 Sep 9;11(9):615. doi: 10.3390/metabo11090615 (PMC8465815; doi:10.3390/metabo11090615)
Supplement: Supplementary file 1 [file metabolites-11-00615-s001.zip › metabolites-1326933-supplementary.pdf]

metabolites

MDPI

Supplementary Materials

Table 1. Correlations overview between metabolites and CRP at all 3 time points.

Time point	Metabolite class	CRP (pre- LVAD)	Time point	Metabolite class	CRP (30 days post- LVAD)	Time point	Metabolite class	CRP (100 days post- LVAD)
(pre- LVAD	AC	1	30 days post-LVAD	AC	4	100 days post-LVAD	AC	0
	AA	1		AA	1		AA	1
	BA	1		BA	2		BA	0
	PC	12		PC	21		PC	1
	SM	0		SM	6		SM	2

Correlated were the metabolites of the individual metabolite class with the respective CRP value. Two- tailed significance was calculated for pre-LVAD. One- tailed significance was calculated for 30days post-LVAD and >100days post-LVAD. AC= Acylcarnitine, AA=Aminoacids, BA= Biogenic amines, PC= Phosphatidylcholines, SM=Sphingomyelins.

Table 2. Correlations overview between metabolites and B at all 3 time points.

Time point	Metabolite class	CRP (pre- LVAD)	Time point	Metabolite class	CRP (30 days post- LVAD)	Time point	Metabolite class	CRP (100 days post- LVAD)
(pre- LVAD	AC	9	30 days post-LVAD	AC	8	100 days post-LVAD	AC	0
	AA	0		AA	0		AA	0
	BA	0		BA	2		BA	0
	PC	9		PC	6		PC	20
	SM	0		SM	0		SM	1

Correlated were the metabolites of the individual metabolite class with the respective BNP value. Two- tailed significance was calculated for pre-LVAD. One- tailed significance was calculated for 30days post-LVAD and >100days post-LVAD. AC= Acylcarnitine, AA=Aminoacids, BA= Biogenic amines, PC= Phosphatidylcholines, SM=Sphingomyelins.

Table 3. Detailed correlations and significances.

Time point	Metabolice class	Metabolite	Laboratory parameter	Spearmans ϱ	Significance *p*
pre-LVAD	AC	C5-OH_C3-DC-M	CRP	0.502	0.001
	AA	Gln		-0.345	0.032
	BA	Kynurenine		0.473	0.002
	PC	lysoPC_a_C17_0		-0.350	0.029
		PC_aa_C32_2		-0.335	0.037
		PC_aa_C34_4		-0.341	0.034
		PC_aa_C36_3		-0.405	0.011
		PC_aa_C36_5		-0.318	0.049
		PC_aa_C38_5		-0.349	0.03
		PC_ae_C34_2		-0.422	0.007
		PC_ae_C34_3		-0.382	0.016
		PC_ae_C36_2		-0.365	0.022
		PC_ae_C36_3		-0.386	0.015
		PC_ae_C38_0		-0.323	0.045
		PC_ae_C40_1		-0.341	0.034
	SM			no correlations	
	AC	C12_DC		0.372	0.02
		C12_1		0.423	0.007
		C14_1		0.377	0.018
		C14_2		0.417	0.008

pre-LVAD		C18_1_OH	BNP	0.326	0.043
		C3_DC_C4_OH		0.390	0.014
		C5_M_DC		0.395	0.013
		C5_OH_C3_DCM		0.357	0.026
		C6_C4_1_DC		0.555	<0.001
	AA			no correlations	
	BA			no correlations	
	PC	lysoPC_a_C18_2		-0.369	0.021
		PC_aa_C32_2		-0.348	0.03
		PC_aa_C34_3		-0.327	0.042
		PC_aa_C34_4		-0.404	0.011
		PC_aa_C36_3		-0.374	0.019
		PC_aa_C36_6		-0.407	0.01
		PC_ae_C36_2		-0.317	0.05
		PC_ae_C38_0		-0.385	0.015
		PC_ae_C40_1		-0.383	0.016
30days post-LVAD	AC	C12_1	CRP	0.306	0.027
		C2		0.286	0.037
		C3_DC_C4_OH		0.280	0.04
		C5_M_DC		0.286	0.037
	AA	His		0.270	0.046
	BA	Kynurenine		0.347	0.014
		Total_DMA		0.405	0.005
	PC	lysoPC_a_C17_0		-0.565	<0.001
		lysoPC_a_C18_2		-0.532	<0.001
		PC_aa_C28_1		-0.374	0.009
		PC_aa_C30_0		-0.306	0.027
		PC_aa_C32_2		-0.283	0.038
		PC_aa_C32_3		-0.298	0.031
		PC_aa_C34_3		-0.439	0.002
		PC_aa_C34_4		-0.601	<0.001
		PC_aa_C36_0		-0.430	0.003
		PC_aa_C36_2		-0.331	0.019
		PC_aa_C36_3		-0.472	0.001
		PC_aa_C36_5		-0.418	0.004
30days post-LVAD	PC	PC_aa_C36_6	CRP	-0.571	<0.001
		PC_aa_C38_5		-0.493	0.001
		PC_ae_C34_3		-0.268	0.047
		PC_ae_C36_1		-0.284	0.038
		PC_ae_C36_4		-0.499	0.001
		PC_ae_C36_5		-0.376	0.008
		PC_ae_C38_0		-0.551	<0.001
		PC_ae_C38_3		-0.417	0.004
		PC_ae_C40_1		-0.530	<0.001
	SM	SM_OH_C14_1		-0.370	0.009
		SM_OH_C22_1		-0.412	0.004
		SM_OH_C22_2		-0.291	0.034
		SM_C16_1		-0.321	0.022
		SM_C18_1		-0.269	0.047
		SM_C24_0		-0.341	0.016
		C12_1		0.455	0.002
		C14		0.398	0.006
		C14_1		0.444	0.002
		C14_2		0.513	<0.001

30days post-LVAD	AC	C18_1_OH	BNP	0.370	0.01
		C2		0.348	0.015
		C3_DC_C4_OH		0.391	0.007
		C5MDC		0.395	0.006
	AA			no correlations	
	BA	Kynurenine		0.292	0.036
		Total_DMA		0.477	0.001
	PC	lysoPC_a_C18_2		-0.364	0.01
		PC_aa_C34_4		-0.404	0.005
		PC_aa_C36_5		-0.337	0.018
		PC_aa_C36_6		-0.370	0.01
		PC_ae_C36_4		-0.273	0.047
		PC_ae_C40_1		-0.345	0.016
	SM			no correlations	
>100days post-LVAD	AC		CRP	no correlations	
	AA	His		-0.291	0.043
	BA			no correlations	
	PC	lysoPC_a_C18_2		-0.339	0.022
	SM	SM_OH_C14_1		0.287	0.045
		SM_C16_0		0.287	0.045
>100days post-LVAD	AC		BNP	no correlations	
	AA			no correlations	
	BA			no correlations	
	PC	lysoPC_a_C18_2		-0.562	0.002
		PC_aa_C30_0		-0.382	0.03
		PC_aa_C32_2		-0.478	0.008
		PC_aa_C32_3		-0.582	0.001
		PC_aa_C34_2		-0.521	0.004
		PC_aa_C34_3		-0.628	<0.001
		PC_aa_C34_4		-0.488	0.007
		PC_aa_C36_2		-0.540	0.003
		PC_aa_C36_3		-0.474	0.008
		PC_aa_C36_5		-0.359	0.039
		PC_aa_C36_6		-0.380	0.03
		PC_ae_C30_2		-0.342	0.047
		PC_ae_C32_2		-0.387	0.028
		PC_ae_C34_3		-0.416	0.019
		PC_ae_C36_2		-0.457	0.011
		PC_ae_C36_3		-0.468	0.009
		PC_ae_C38_0		-0.348	0.044
>100days post-LVAD	PC	PC_ae_C38_3	BNP	-0.387	0.028
		PC_ae_C40_1		-0.442	0.014
		PC_ae_C42_2		-0.458	0.011
	SM	SM_C16_1		-0.380	0.03

Correlated were the metabolites with the respective CRP or BNP value. Two- tailed significance was calculated for pre-LVAD. One- tailed significance was calculated for 30days post-LVAD and >100days post-LVAD. AC= Acylcarnitine, AA=Aminoacids, BA= Biogenic amines, PC= Phosphatidylcholines, SM=Sphingomyelins.
